# Repositioning Fluoxetine as a TRPV3 Channel Inhibitor to Alleviate Skin Inflammation and Pruritus

**DOI:** 10.3390/cimb47040277

**Published:** 2025-04-15

**Authors:** Ling Zhang, Junjie Chang, Yimei Xu, Qi Ge, Congxiao Zhang

**Affiliations:** Department of Pharmacology, School of Pharmacy, Qingdao Medical College, Qingdao University, Qingdao 266073, China; zhangling@qdu.edu.cn (L.Z.); junjiechangyl@163.com (J.C.); 2020021135@qdu.edu.cn (Y.X.); geqi1113@163.com (Q.G.)

**Keywords:** drug repurposing, TRPV3, fluoxetine, skin inflammation

## Abstract

Transient receptor potential vanilloid 3 (TRPV3) is a non-selective cation channel prominently present in the skin. It plays a role in diverse physiological and pathological functions like inflammation of the skin, pain sensations in the skin, and persistent itchiness. Overactive TRPV3 channels contribute to numerous inflammatory skin diseases, and this highlights the therapeutic potential of its inhibitors. Using a drug repurposing screening approach, we identified fluoxetine—a clinically established antidepressant agent—as a potent inhibitor of TRPV3 channel activation, demonstrating its therapeutic potential for skin inflammation alleviation. During whole-cell patch-clamp recordings, fluoxetine exhibits a selective inhibitory effect on macroscopic TRPV3 currents in a concentration-dependent fashion. The IC_50_ value is measured as 10.23 ± 2.34 μM. On the single-channel scale, fluoxetine leads to a reduction in both single-channel conductance and the open probability of the channel. In the course of animal experiments, fluoxetine mitigates carvacrol-induced TRPV3-related skin inflammation. It lessens the severity of dorsal lesions and ear edema in mice. Our study not only identified TRPV3 as a novel target of fluoxetine and provides new ideas for the treatment of TRPV3-mediated skin diseases with fluoxetine, but also provides a valuable tool molecule for further understanding TRPV3 channel pharmacology.

## 1. Introduction

Transient receptor potential vanilloid 3 (TRPV3), a calcium-permeable temperature-sensitive cation channel, is abundantly expressed in the skin keratinocytes, dorsal root ganglia, and nasal and oral epithelial cells [1,2,3,4]. Activation of TRPV3 increases calcium ion permeability across the cell membrane. This induces a rapid influx of extracellular calcium ions, leading to a significant increase in intracellular calcium concentration. The elevated calcium concentration, functioning as a critical second messenger, triggers downstream signaling cascades. For example, elevated calcium levels enhance the responsiveness of human keratinocytes to Th2-mediated inflammatory stimuli via NF-κB activation [5]. TRPV3 interacts with transglutaminase to form a functional complex that modulates growth factor receptor signaling, thereby regulating keratinocyte proliferation, differentiation, and skin barrier homeostasis. Dysregulated TRPV3 activation disrupts the balance between keratinocyte proliferation and differentiation, ultimately impairing skin barrier function [6,7]. Overactivity of TRPV3 function has been implicated in the pathology of Olmsted syndrome [8], chronic pruritus [9,10,11], alopecia [12,13] and dermatitis [14,15,16]. All these observations suggest that the identification of effective TRPV3 inhibitors will have great significance for the treatment of related diseases.

In the past few years, a number of plant-derived compounds have been identified as TRPV3 channel inhibitors. These compounds include osthole, forsythoside B, and the isomers isochlorogenic acid A and isochlorogenic acid B. Osthole exerts an inhibitory effect on TRPV3 through allosteric competitive inhibition, reduces the expression of inflammatory factors TNF-α and IL-6 in animal atopic dermatitis models, and alleviates pruritus in the skin of AD mice. Forsythoside B can inhibit skin inflammation and pruritus by inhibiting the NF-kB signaling pathway. Isochlorogenic acids A and B showed the ability to alleviate ear swelling and pruritus in mice by directly inhibiting TRPV3 [17,18,19]. Although they have shown therapeutic effects in animal experiments, the lack of studies on features such as bioavailability limits their clinical translation process. This limitation highlights the need for alternative strategies to identify TRPV3 inhibitors with established pharmacological profiles. Recent studies indicate that the local anesthetic dyclonine effectively inhibits TRPV3 channels, revealing a molecular mechanism [20,21]. Through FDA-approved drug screening, researchers identified the antispasmodic flopropione as a selective TRPV3 inhibitor capable of alleviating skin inflammation [22].

Unlike traditional drug discovery, drug repurposing has emerged as a promising means for expediting the drug discovery process [23]. Having prior knowledge about safety and efficacy routes of administration can significantly reduce development costs and shorten development time, thereby reducing the effort required to successfully bring a repositioned drug to market [24]. Therefore, we directed our research focus toward drug repurposing with the goal of identifying novel TRPV3 inhibitors, aiming to provide potential therapeutic strategies for TRPV3-related diseases and advance the pharmacological understanding of this channel. Through screening of an FDA-approved drug library, we successfully identified fluoxetine (FLX) as a potent TRPV3 inhibitor. Fluoxetine is a kind of selective serotonin (5-HT) reuptake inhibitor (SSRI) antidepressant. Approved by the FDA in 1987, fluoxetine mainly inhibits the reuptake of serotonin (5-HT) by the presynaptic membrane, increasing the concentration of 5-HT in the synaptic cleft and enhancing serotonergic neurotransmission, thus exerting its antidepressant effect. Additionally, it is frequently employed in dealing with psychiatric conditions like obsessive–compulsive disorder and anxiety disorder, as well as metabolic disorders [25,26,27].

This study reveals that fluoxetine powerfully inhibits human TRPV3 channel currents. Preliminary mechanistic studies revealed that fluoxetine modulates both channel conductance and gating properties, while in animal models it has demonstrated significant anti-pruritic and anti-inflammatory effects. This discovery not only highlights fluoxetine’s repurposing potential but also establishes it as a key pharmacological tool for probing TRPV3 channel pharmacology.

## 2. Materials and Methods

### 2.1. Chemicals

Fluoxetine (MW: 309.33), dexamethasone (MW: 392.46), capsaicin (MW: 305.41) and A-967079 (MW: 207.24) compounds were procured from TargetMol (Shanghai, China). Compounds TRPV3 agonist 2-aminoethoxydiphenyl borate (2-APB), TRPV3 agonist carvacrol, TRPV4 agonist GSK1016790-A (GSK101), TRPA1 agonist allyl isothiocyanate (AITC) and TRPM8 agonist menthol were procured from Sigma-Aldrich (St. Louis, MO, USA). Prior to utilization, the compounds were prepared as stock solutions in DMSO. The compounds used for patch-clamp recordings were diluted within the perfusion solution. To establish dermatitis and ear swelling models, 3% carvacrol was diluted in a 30% ethanol solution (*v*/*v*) solvent before topical application. This solvent-dilution step ensured proper delivery of the carvacrol for model induction. The compounds utilized for treatment were diluted in a solvent of saline containing 50% PEG300.

### 2.2. Cell Cultures and Transient Transfection

The HEK293T cell line was obtained from the Cell Resource Center, Peking Union Medical College (Beijing, China). HEK293T cells were cultivated in Dulbecco’s modified Eagle’s medium (DMEM, Gibco, ThermoFisher, Grand Island, NY, USA), fortified with 10% fetal bovine serum (FBS, PAN-Biotech, Aidenbach, Bayern, Germany). The cell culture was incubated at 37 °C with 5% CO_2_. Twenty-four hours prior to transfection, HEK293T cells were treated with trypsin and seeded onto glass cover slips. For transfection, 2 μg of cDNAs corresponding to human TRPV3 (hTRPV3, NM_145068.4), hTRPV1 (NM_080704.3), hTRPV4 (NM_021625.5), hTRPA1 (NM_007332.3), hTRPM8 (NM_024080.5) and mouse TRPV3 (mTRPV3, NM_145099) were transiently introduced into cells within a 35 mm cell culture dish. For single-channel recordings, 0.2 μg of human TRPV3 cDNAs were transfected. Lipofectamine 2000 (Invitrogen, Carlsbad, CA, USA ) was used for this transfection process, strictly following the manufacturer’s instructions. Transfected cells were identified based on the expression of enhanced green fluorescent protein (EGFP), which served as a green fluorescence marker. Subsequently, electrophysiological recordings could be carried out.

### 2.3. Electrophysiology

Whole-cell patch-clamp recordings were conducted with an EPC10 amplifier that was driven by Patch Master software (v2x90.5) (HEKA Harvard, Holliston, Church Hill, TN, USA). A DMZ universal electrode puller (PC-100, Narishige, Tokyo, Japan) was utilized to draw the borosilicate glass pipettes to a resistance of 3–6 MΩ. The pipette solution as well as the bath solution each consisted of (in mM) 130 NaCl, 3 HEPES, and 0.2 EDTA, with a pH value of 7.4. The cell membrane potential was held steady at 0 mV. A voltage ramp spanning from −100 mV to +100 mV over a duration of 500 ms was applied, during which currents were recorded. Subsequently, these currents were analyzed at both +80 mV and −80 mV.

The MultiClamp 700B amplifier, regulated by Clampex 11.0.3 software, was utilized for single-channel recordings. Borosilicate glass capillaries were used to create borosilicate glass pipettes, with resistances falling in the range of 6–10 MΩ. For inside-out single-channel recordings, the composition of the pipette and bath solutions was as follows (in mM): 130 NaCl, 3 HEPES, and 0.2 EDTA, adjusted to pH 7.4. The membrane potential was set at −60 mV. Currents were digitized at a frequency of 10 kHz and filtered at 3 kHz.

All patch-clamp experiments were carried out at room temperature (22 ± 2 °C).

In the in vitro experiments, we referred to the literature regarding the screening and identification of TRPV3 inhibitors. Electrophysiological and CCK-8 cell viability tests (Figure S1) guided our choice. A 0.1–500 μM range was selected as it showed significant effects without excessive cell toxicity.

### 2.4. Animals

Animal experiments were conducted in accordance with the national and institutional guidelines for the care and use of laboratory animals (protocol code 20240504C576020240603034), approval date 10 April 2024 (Qingdao, China). Male black C57BL/6 mice were procured from Beijing Vital River Laboratory (Beijing, China). Mice were 6–8 weeks old and weighed approximately 20 ± 2 g. Mice were housed in the standard laboratory conditions with appropriate room temperature (22 ± 2 °C) and sufficient water and food under a normal circadian rhythm. Upon arrival at the laboratory, mice were acclimated to the new environment for a week before experiments. Upon completion of the experiment operation and data recording, all the mice were put under anesthesia with 1–1.5% isoflurane by means of an isoflurane gas anesthesia apparatus. Subsequently, they were euthanized by cervical dislocation while still in an anesthetized state.

For the animal experiments, we reviewed the literature on mouse dermatitis drug doses. Small-scale preliminary animal tests were carried out [22]. After observing behavior, dermatitis symptoms and body weight in different dose groups, we set 0.1–10 mM for formal experiments, which effectively treated dermatitis while ensuring safety.

### 2.5. Carvacrol-Induced Mouse Dermatitis and Treatment

Carvacrol, a skin sensitizer, sets off the activation of TRPA1 and TRPV3 channels, triggering intense inflammation in the skin [28]. To generate the carvacrol-induced mouse dermatitis model, prior to establishing the dorsal skin inflammation model, mice were placed in a gas anesthesia apparatus (SurgiVet). Their dorsal hair was shaved, and they were allowed a 24-h period to adapt to the environment. Carvacrol was dissolved in saline solution containing 30% ethanol. Then, 100 μL of 3% carvacrol was topically applied to the dorsal skin of mice once daily for 5 consecutive days. In the blank control group (Control), mice received a daily topical application of 100 μL of 30% ethanol solution on their dorsal skin for 5 consecutive days. In the model group (Carvacrol), 100 μL of 10% DMSO solution (with the solvent being physiological saline containing 50% PEG300) was applied to the dorsal skin of mice 30 min prior to the topical application of carvacrol. For the treatment groups with different doses of fluoxetine, 100 μL of fluoxetine solutions at concentrations of 0.1 mM, 1 mM and 10 mM were, respectively, applied to the dorsal skin of mice 30 min before the topical application of carvacrol, once daily. The positive control treatment group was applied with an equal volume of 10 mM dexamethasone solution at the same time point. On the 5th day after the application of carvacrol, the level of pruritus was determined by counting the number of scratching bouts within 15 min. A scratching bout of the mouse consisted of a series of actions where it lifted its hind limb towards the dorsal skin and scratched for a certain period until the limb moved back to the ground or into its mouth. On the 6th day, the mice were euthanized under anesthesia, and then skin samples were collected and fixed with 4% paraformaldehyde. All animal experiments were evaluated by individuals blinded to the treatment conditions.

### 2.6. Carvacrol-Induced Mouse Ear Swelling and Treatment

The process is identical to the dorsal model mentioned above without shaving; the solvent (30% ethanol solution) was used as a control. Three doses of fluoxetine (0.1 mM, 1 mM, 10 mM; 100 μL each) or a 10% DMSO vehicle solution (saline with 50% PEG300) were topically applied to mouse ears daily. Each application occurred 30 min prior to the topical administration of carvacrol. Similarly, the same volume of Dex (10 mM) was used as a positive control.

### 2.7. Skin Lesion Scoring and Ear Thickness Measurements

For the composite dermatitis score, dermatitis severity was assessed based on the following four symptoms: (1) erythema/hemorrhage; (2) scarring/dryness; (3) edema; and (4) excoriation/erosion. The score for each symptom ranged from 0 to 3 (0, none; 1, mild; 2, moderate; and 3, severe) [16]. Total score is defined as the sum of individual scores, with a range of 0~12. Prior to the treatment with fluoxetine or dexamethasone, over five consecutive days, the thickness of the mice’s ears was measured daily with a Vernier caliper.

### 2.8. Histopathological Examination

Mouse back skin and ears were excised and fixed in 4% paraformaldehyde overnight. Subsequently, they were embedded in paraffin and sliced to a thickness of 2 microns. They were then stained with hematoxylin and eosin (H&E). Images were acquired with a bright-field microscope (ECLIPSE Ti, Nikon, Tokyo, Japan) fitted with a CCD camera (DS-Ri2, Nikon, Japan). The epidermal thickness was gauged using the linear distance function within the NIS-Elements BR software (Version 4.60.00(Build 1171)LO,64bit) (DS-Ri2, Nikon, Japan).

### 2.9. Statistical Analysis

All data are presented as mean ± standard deviation (SD). Statistical significance was assessed using paired *t*-tests and one-way or two-way analysis of variance (ANOVA), followed by multiple-comparison tests. Igor Pro (Wave-metrics, Portland, OR, USA), GraphPad Prism 8.0 (GraphPad Software, Boston, MA, USA) and Origin64 (OriginLab, Northampton, MA, USA) software were utilized for these analyses. A *p*-value of less than 0.05 was deemed statistically significant.

## 3. Results

### 3.1. Identification of Fluoxetine as a TRPV3 Inhibitor in Patch-Clamp Recordings

**Figure 1:**
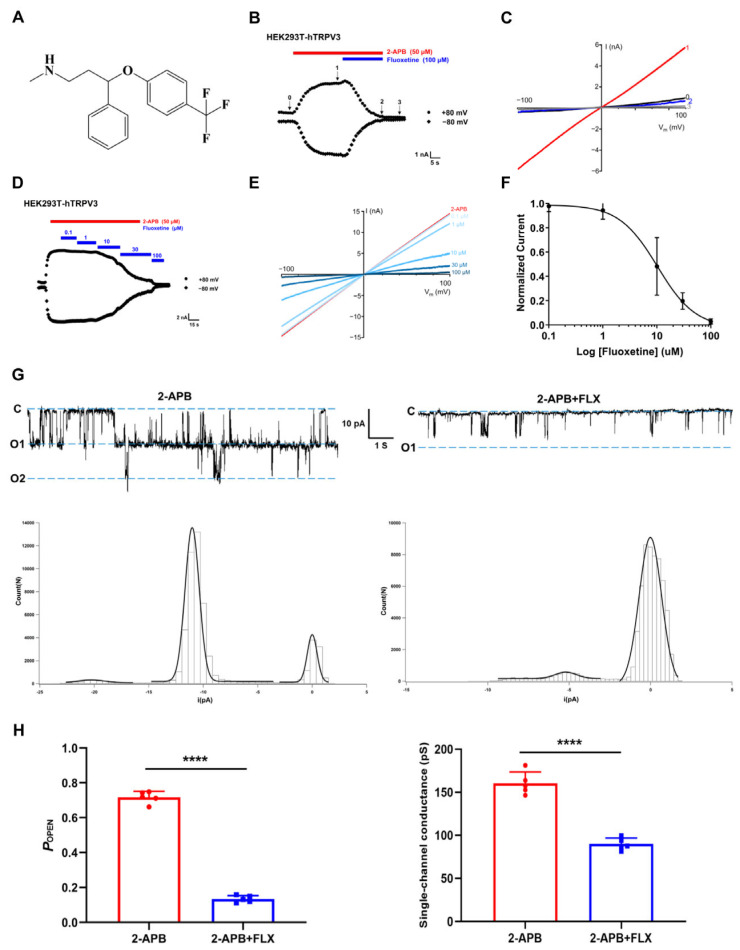
Fluoxetine effectively suppresses TRPV3 currents induced by 2-APB. (**A**) the molecular structure of fluoxetine. (**B**) Whole-cell patch-clamp recordings of TRPV3 channel currents in HEK293T cells, comparing the response to 2-APB alone (50 μM, indicated by the red bar) with the combined application of 100 μM fluoxetine (blue bar). (**C**) Current–voltage curves of TRPV3, measured during voltage ramps ranging from −100 mV to +100 mV. Data were collected at four stages: baseline (0), after administering 50 μM 2-APB (1), during the simultaneous application of 50 μM 2-APB and 100 μM fluoxetine (2), and following washout (3) (**D**) Whole-cell currents were progressively suppressed by fluoxetine concentrations ranging from 0.1 to 100 μM. (**E**) voltage ramps from −100 to +100 mV were used to generate current–voltage curves for TRPV3, both in the presence of 50 μM 2-APB and in combination with varying fluoxetine concentrations. (**F**) The dose-dependent inhibition of 2-APB-induced TRPV3 activation by fluoxetine was analyzed using the Hill equation, with results expressed as mean ± SD. (**G**) Single-channel recordings of TRPV3 currents were obtained in an inside-out configuration, either with 30 μM 2-APB alone or alongside 50 μM fluoxetine. The dotted lines in the left panels mark the open channel state. The right panel shows all point amplitude histograms for the single-channel open (O) and closed (C) states. (**H**) A summary of hTRPV3 single-channel open probability (POPEN) and conductance values is provided, comparing conditions with and without 2-APB or fluoxetine. *n* = 5, **** *p* < 0.0001. Data are shown as the mean ± SD, paired *t*-test.

Taking the maximum current of the TRPV3 channel evoked by 2-APB and the basal current after washout as reference, we found that the co-administration of fluoxetine at a concentration of 100 μM decreased the 2-APB-induced TRPV3 current by 92.3 ± 2.5% (*n* = 5) (Figure 1A–C). Furthermore, we found that fluoxetine inhibited 2-APB-evoked TRPV3 currents in a dose-dependent manner. We recorded the changes in the current after administering fluoxetine at different concentrations. Then, we calculated the inhibition rate of the current of the TRPV3 channel by fluoxetine at different concentrations. We used Origin software to perform Hill equation fitting and determined that the half maximal inhibitory concentration (IC_50_) value was 10.23 ± 2.34 μM. (*n* = 5) (Figure 1D–F).

To further confirm the direct effect of fluoxetine on single TRPV3 channels, we also tested how fluoxetine (50 μM) affects single-channel currents evoked by 2-APB (30 μM) using inside-out configurations (Figure 1G). In inside-out patches excised from hTRPV3-expressing HEK293T cells, at a concentration of 50 μM, fluoxetine inhibited the 2-APB-induced current, as manifested by a reduction in the channel open probability (NPo) at −60 mV, with NPo dropping from 0.71 ± 0.03 to 0.13 ± 0.01 (*n* = 5, *p* < 0.0001) and simultaneously the single-channel conductance decreasing from 160.36 ± 13.24 pS to 93.56 ± 14.37 pS (*n* = 5, *p* < 0.0001) (Figure 1H). These results indicate that fluoxetine may inhibit channel activation via a pore-occluding mechanism. Collectively, our findings reveal that fluoxetine functioned as an inhibitor of TRPV3.

### 3.2. Selectivity of Fluoxetine Among Thermo-TRP Channels

**Figure 2:**
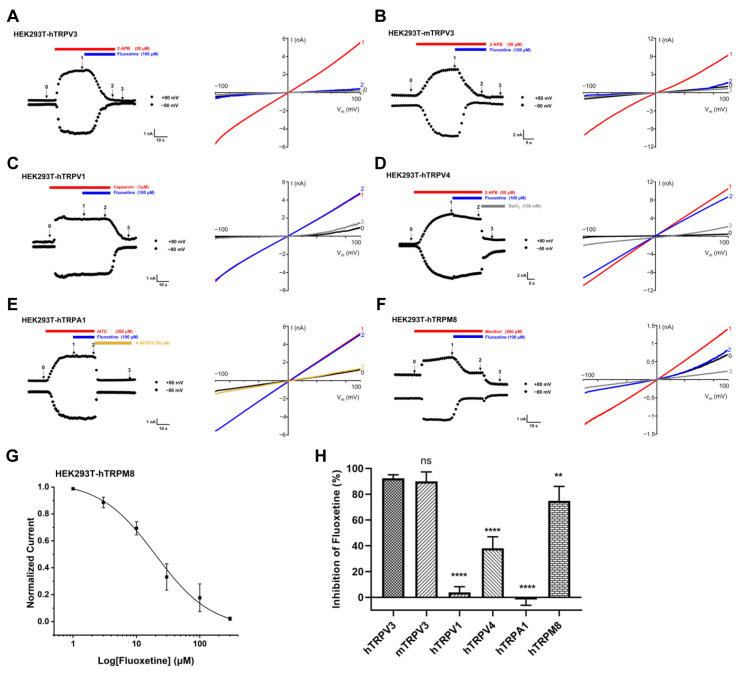
Selective inhibition of whole-cell TRPV3 currents by fluoxetine over other TRP channels. (**A**) Whole-cell current of hTRPV3 in response to 50 μM 2-APB, and co-application of 100 μM fluoxetine displaying obvious inhibition on hTRPV3. (**B**) Whole-cell current of mTRPV3 in response to 50 μM 2-APB, and co-application of 100 μM fluoxetine displaying obvious inhibition on mTRPV3. (**C**) Whole-cell current of hTRPV1 in response to 1 μM capsaicin, and co-application of 100 μM fluoxetine. (**D**) Whole-cell current of hTRPV4 in response to 1 μM GSK1016790A, and co-application of 100 μM fluoxetine. (**E**) Whole-cell current of hTRPA1 in response to 300 μM AITC, and co-application of 100 μM fluoxetine. (**F**) TRPM8 currents recorded by perfusing cells with a bath solution containing 500 μM menthol to evoke the currents and observe the effect of fluoxetine on these currents. (**A**–**F**) Each right plate corresponds one-to-one to the left plate, showing current–voltage curves of the corresponding channel recorded during voltage gradient from −100 mV to + 100 mV and measured initially (0), at agonist administration (1), on co-application with 100 μM fluoxetine (2), and after the washout (3). (**G**) Fitting of the Hill equation was carried out to analyze the dose-dependent inhibition of menthol-induced TRPM8 activation by fluoxetine. Data are presented as the mean ± SD. (**H**) Summary of inhibition of hTRPV3, mTRPV3, hTRPA1, hTRPV1 and hTRPV4 currents by 100 μM fluoxetine. Data are shown as the mean ± SD; *n* = 5; ** *p* < 0.01, **** *p* < 0.0001, ns, no significance by one-way ANOVA, followed by Dunnet’s test.

To further determine its selectivity, we tested the inhibition rate of fluoxetine on other dermatitis-related TRP channels transiently expressed in HEK293T cells, such as TRPV1, TRPV4, TRPA1, and TRPM8. When compared to the positive control where 1 μM agonist capsaicin activated TRPV1, 100 μM fluoxetine exerted no impact on TRPV1. Similarly, in contrast to the positive agonist control of 300 μM AITC which activated TRPA1 or the positive inhibitor control of 50 μM A-967079, 100 μM fluoxetine had no effect on TRPA1. Fluoxetine at 100 μM had a slight inhibition of GSK101-evoked TRPV4 currents by 14.32 ± 9.73%. However, when testing the effect of 100 μM fluoxetine on TRPM8, we observed an approximately 70% inhibition of TRPM8 currents activated by menthol at 500 μM (Figure 2A–E). In addition, we determined the dose-dependent inhibition of TRPM8 currents by fluoxetine with an IC_50_ value of 20.0 ± 3.3 μM (Figure 2F). In addition, we determined that 100 μM Fluoxetine has the same inhibition effect on the 2-APB-evoked mouse TRPV3 currents. After statistical analysis, we can come to a conclusion: fluoxetine exhibited good selectivity for TRPV3 (Figure 2G).

### 3.3. Fluoxetine Alleviates Carvacrol-Induced Dermatitis and Pruritus

**Figure 3:**
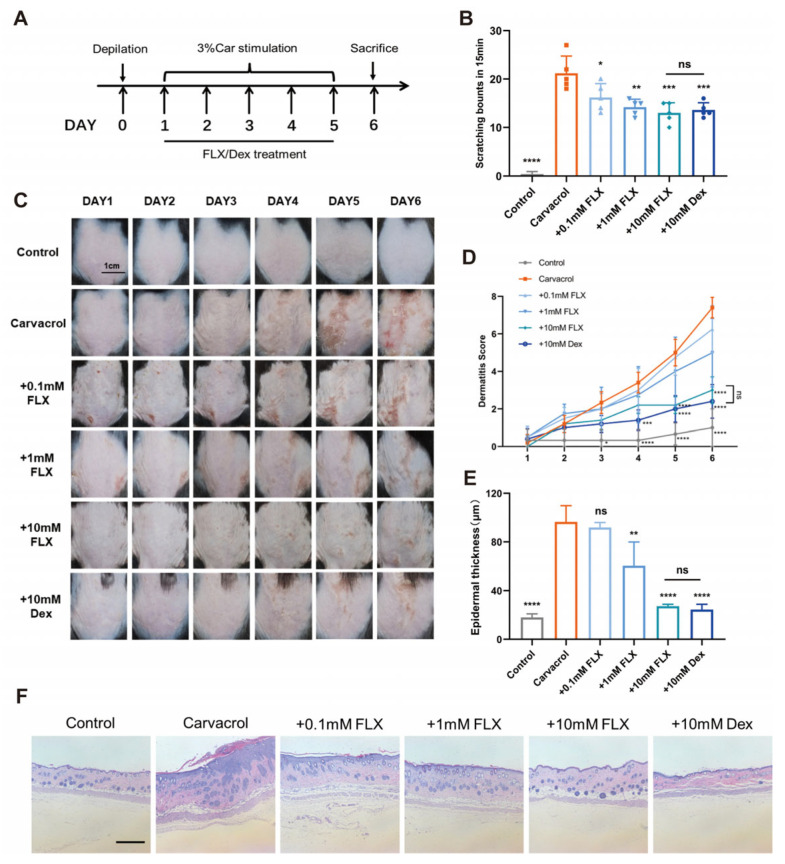
Fluoxetine alleviates carvacrol-induced dermatitis and pruritus. (**A**) A flowchart depicting the establishment of carvacrol-induced mouse dermatitis and the administration of fluoxetine (FLX) and dexamethasone (Dex). (**B**) Quantification of scratching bouts in mice treated with Veh, Car, Car plus FLX (0.1 mM, 1 mM, 10 mM) and Car plus Dex (10 mM) (*n* = 5, * *p* < 0.05, ** *p* < 0.01, *** *p* <0.001, **** *p* < 0.0001, ns, no significance. by one-way ANOVA, followed by Dunnett’s multiple comparisons test). (**C**) Phenotypic features of mouse skin before and after 3% carvacrol in the presence of different concentrations of FLX or Dex for six consecutive days. Scale bar = 1 cm. (**D**) Dermatitis scores from panel C (*n* = 5, * *p* <0.05, *** *p* < 0.001, **** *p* < 0.0001, ns, no significance, by two-way ANOVA, followed by Dunnett’s multiple comparisons test). All data are presented as the mean ± SD. (**E**) Statistical analyses performed on the thicknesses of dorsal skin sections from various mouse groups (*n* = 5). ** *p* < 0.01, **** *p* < 0.0001, ns, no significance, by one-way ANOVA, followed by Dunnet’s test. All data are expressed as the means ± SD. (**F**) Presentative histological H&E staining images of 6 μm paraffin-embedded sections of mouse dorsal skin are shown. The images were taken before and after carvacrol treatment or after treatment with different concentrations of fluoxetine or dexamethasone. Scale bar = 500 μm.

The over-activation of TRPV3 induces ion channel disease in humans and rodents. On the basis of previous experiments [16], we set up dermatitis induced by TRPV3 agonist carvacrol. In the present study, we used a mouse dermatitis model produced by the topical application of 3% carvacrol, and we used different concentrations of fluoxetine and the effective drug dexamethasone to treat carvacrol-induced skin inflammation in mice with dermatitis (Figure 3A). Carvacrol treatment induced pruritus, as manifested by the increased number of scratching bouts from 0.4 ± 0.5 to 21.2 ± 3.6 per 15 min (*n* = 5, *p* < 0.0001), and fluoxetine dose-dependently decreased the pruritus (Figure 3B). As shown in the typical phenotype images in mice, 3% carvacrol induced erythema with inflamed and scaly skin (Figure 3C). On the other hand, fluoxetine applied topically at various concentrations ranging from 0.1 to 10 mM mitigated skin inflammation in a concentration-related fashion (Figure 3C) and significantly reduced the dermatitis scores, as compared with the carvacrol group (Figure 3D). Moreover, as shown by the increased epidermal thickness in H&E staining, topical application of fluoxetine alleviated inflammation in the back skin of mice compared with the model group (Figure 3E,F). Meanwhile, there was no significant difference between the 10 mM fluoxetine group and the dexamethasone group in terms of skin inflammation score, epidermal thickness and amount of scratching (*p* > 0.05), indicating that fluoxetine can show similar efficacy to dexamethasone.

### 3.4. Fluoxetine Alleviates Carvacrol-Induced Ear Swelling

**Figure 4:**
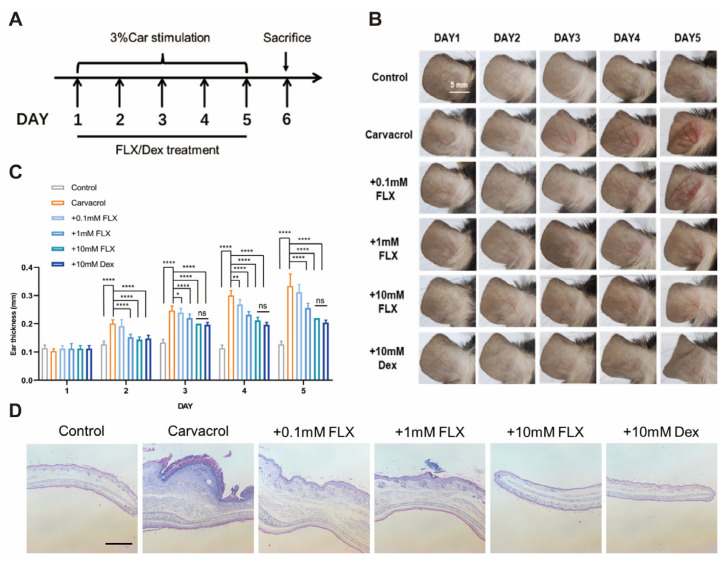
Attenuation of carvacrol-induced ear swelling by topical fluoxetine. (**A**) A flowchart depicting the establishment of carvacrol-induced mouse ear swelling and the administration of fluoxetine (FLX) and dexamethasone (Dex). (**B**) Observation for 5 consecutive days in representative ear images of mice before the administration of FLX and Dex every day. (**C**) Summary of ear thickness for carvacrol-induced ear swelling in mice treated with fluoxetine at different concentrations and dexamethasone. Statistical significance was analyzed using two-way ANOVA (*n* = 5). * *p* < 0.05, ** *p* < 0.001, **** *p* < 0.0001, ns, no significance. Data are presented as the means ± SD. (**D**) Representative histological images from H&E-stained paraffin-embedded sections (6 μm thick) of mouse ears are presented. These show the ears before and after treatment with carvacrol, as well as when exposed to different concentrations of fluoxetine and dexamethasone. Scale bar = 500 μm.

We also assessed the effect of fluoxetine on ear swelling (Figure 4A). Local application of 3% carvacrol led to a significant increase in the degree of ear swelling in mice. Conversely, when fluoxetine at varying concentrations was topically applied over a 4-day period, it remarkably alleviated the redness and swelling of the ears (Figure 4B,C).

Subsequent histological examinations were conducted on ear tissue sections. The findings revealed that, in comparison to the group treated with carvacrol alone (Figure 4D), topical application of fluoxetine at varying concentrations, as well as dexamethasone, alleviated carvacrol-induced ear swelling in mice. These results align with the phenotypic observations mentioned earlier. In addition, in terms of ear swelling, the 10 mM fluoxetine group was analyzed against the dexamethasone group, and there was no significant difference between the two groups, indicating a comparable treatment effect.

## 4. Discussion

**Mechanistic insights and therapeutic potential of fluoxetine in TRPV3-mediated skin inflammation.** In this study, we screened the FDA-approved drugs to find a drug that is effective on TRPV3 channels, and we identified an antidepressant agent, fluoxetine (FLX). Despite the fact that it shows inhibitory effects on TRPM8 channels, the antidepressant fluoxetine selectively inhibits macroscopic and single-channel TRPV3 currents to a higher degree. Fluoxetine showed a higher selectivity for TRPV3 channel inhibition with an IC_50_ of 10.23 ± 2.34 μM compared to the inhibition of TRPM8 with an IC_50_ of 20.0 ± 3.38 μM. TRPM8, a cold-sensitive ion channel, plays a significant role in itch perception [29]. Previous research has shown that its activation reduces scratching and inflammatory markers [30,31], yet there is a lack of studies on inhibiting TRPM8 in dermatitis. Our electrophysiological experiments revealed that fluoxetine does not activate TRPM8 (Figure S2). Considering these findings, relevant studies, and our additional research, we propose that the anti-inflammatory effect of fluoxetine mainly occurs via TRPV3 regulation. We are aware that precisely delineating the contribution of TRPV3 and TRPM8 to the anti-inflammatory effects of fluoxetine is complex, which is a limitation of the current study and sets the direction for our future research to refine our understanding by employing genetic techniques and other methodologies. In the TRPV3 agonist carvacrol-treated mouse dermatitis and ear-swelling model, fluoxetine effectively reduced skin inflammation, epidermal thickening and ear swelling. The therapeutic effect of fluoxetine was comparable to that of dexamethasone, at least at a concentration of 10 mM. In this study, fluoxetine was repurposed for the treatment of skin inflammation and itching.The therapeutic effects of fluoxetine and another drug–repurposed TRPV3 inhibitor flopropione were evaluated at the animal level, and both of them showed promising therapeutic effects [22] (Figure S3). Topical application of fluoxetine (1–10 mM) could alleviate TRPV3-driven inflammation in mice, with efficacy comparable to that of dexamethasone, suggesting its potential as a steroid-sparing agent.

**Advancing TRPV3 inhibitors through drug repurposing.** Previous studies have reported several structurally distinct TRPV3 inhibitors such as forsythoside B [17], osthole [19], verbascoside [32], isochlorogenic acids A and B [18], scutellarein [33] and hydroxychloroquine [34] and small molecules such as 17(R)-resolvin D1 [35], 26E01 [36], dyclonine [20] and flopropione [22]. At present, the natural products reported have unstable activity, complex structure and low bioavailability, which limit further clinical research. Drug repurposing leveraging existing safety and pharmacokinetic data can effectively reduce the time and money costs of drug research and development. Therefore, the use of approved drugs for studies targeting TRPV3 has the potential to be repurposed. Our discovery of a new target for fluoxetine is an embodiment of this strategy. Fluoxetine has been widely used in the clinical treatment of depression, and there is a relatively abundant amount of safety data available for it. An animal study on fluoxetine’s antidepressant effect showed that at an oral dose of 10 mg/kg administered daily, it took at least 24 days to work [37]. In contrast, our research found that topical fluoxetine achieved anti-dermatitis effects within 5 days with a lower dose. Clinically, oral fluoxetine for depression needs 4–5 weeks to take effect [38]. This time difference suggests that topical application likely exhibits different pharmacokinetics. The quick onset implies local action and less systemic absorption. Unlike long-term oral use, topical fluoxetine does not require continuous intake. Thus, the risk of adverse reactions from drug accumulation during topical dermatitis treatment is low. During the process of clinical translation, several issues regarding fluoxetine need to be addressed. On the one hand, there is a lack of research on the transdermal absorption and pharmacokinetics of fluoxetine. In the further translation of fluoxetine into clinical application, the issues of its transdermal absorption pharmacokinetics should be addressed first and the effective and safe dose of fluoxetine for clinical treatment should be further clarified. On the other hand, to reduce off-target effects such as rashes and gastrointestinal disorders related to the original target, the formulation of fluoxetine could be optimized to increase its transdermal absorption rate while reducing systemic absorption.

## 5. Conclusions

Fluoxetine as a TRPV3 inhibitor has good potential for clinical application in the treatment of dermatological diseases, especially because of its proven safety and cost-effectiveness as an FDA-approved drug. According to our experimental results, topical fluoxetine reduced TRPV3-driven inflammation and pruritus with an efficacy comparable to that of dexamethasone, suggesting that fluoxetine could be used as a steroid-sparing agent for conditions such as atopic dermatitis. Notably, fluoxetine also produces activity against TRPM8 when modulating serotonergic pathways and TRPV3 channels, although target specificity in human skin needs to be evaluated. Overall, further identification of drugs targeting the TRPV3 channel may provide effective treatment options for skin inflammatory diseases. Although the research on fluoxetine as a repurposed drug has achieved certain results, it still faces many challenges and requires further in-depth research and optimization.

## Data Availability

Data are contained within the article and Supplementary Materials. The data that support the findings of this study are available from the corresponding author upon reasonable request (correspondence: zhangcx@qdu.edu.cn).

## Supplementary Materials

The following supporting information can be downloaded at: https://www.mdpi.com/article/10.3390/cimb47040277/s1.

## Abbreviations

5-HT	5-hydroxytryptamine
2-APB	2-aminoethoxydiphenyl borate
AITC	allyl isothiocyanate
DMEM	Dulbecco’s modified Eagle’s medium
DMSO	dimethyl sulfoxide
GSK101	GSK1016790A
H&E	hematoxylin and eosin
HEK293T	human embryonic kidney 293T
SD	standard deviation
