# Supplementary material for: Repositioning Fluoxetine as a TRPV3 Channel Inhibitor to Alleviate Skin Inflammation and Pruritus
Source: Curr Issues Mol Biol. 2025 Apr 15;47(4):277. doi: 10.3390/cimb47040277 (PMC12025681; doi:10.3390/cimb47040277)
Supplement: Supplementary file 1 [file cimb-47-00277-s001.zip › cimb-3563128-supplementary.pdf]

Supplementary information

Repositioning Fluoxetine as a TRPV3 Channel Inhibitor to Alleviate Skin Inflammation and Pruritus

Ling Zhang [1,#], Junjie Chang [1,#], Yimei Xu [1], Qi Ge [1] and Congxiao Zhang [1,*]

[1] Department of Pharmacology, School of Pharmacy, Qingdao Medical College, Qingdao University, Qingdao 266071, China.
# Co-first author.
* Correspondence: zhangcx@qdu.edu.cn

Supplementary Methods

CCK-8 assay

We evaluated the effect of fluoxetine on cell viability using the CCK-8 assay. Seed cells in a 96-well plate (5 × 10^3 cells/well) and cultured overnight. Treat cells with different concentrations of fluoxetine, incubate for 24 hours, then add 10 μL of 10% CCK-8 reagent per well. Incubate for 1 hours until color develops, measure absorbance at 450 nm, and calculate viability using control and blank-corrected OD values.

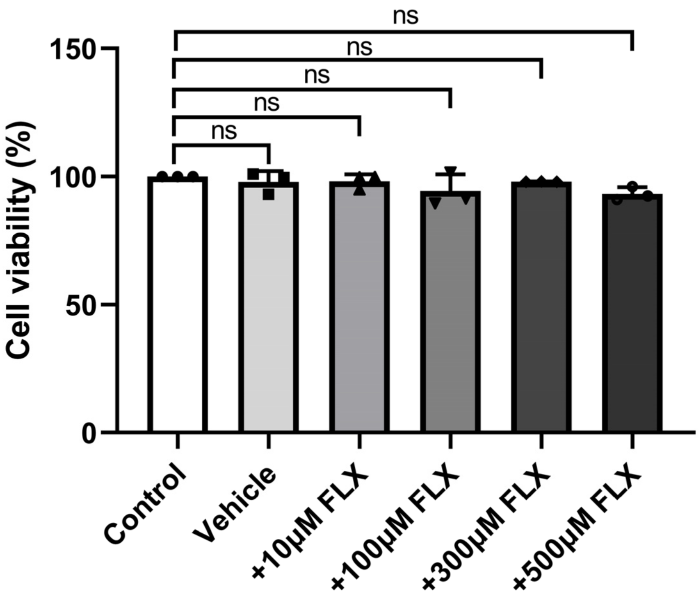

Figure S1. Fluoxetine on cell viability of HEK-293T cells.
The viability of HEK-293T cells was assessed using CCK-8 assay after a 6 h treatment with different concentrations of fluoxetine. Data were expressed as the mean ± SD. n=3, ns, no significance, by one-way ANOVA followed by Dunnett's multiple comparison tests.

Figure S2

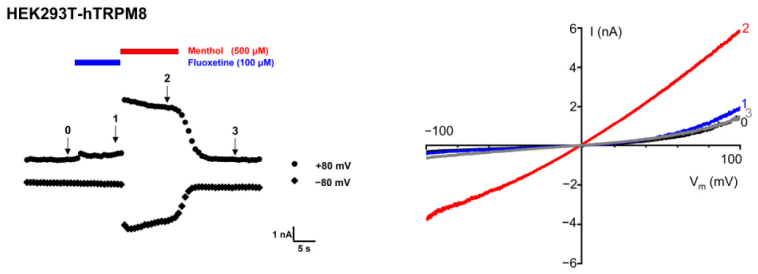

Figure S2. hTRPM8 currents induced by fluoxetine or menthol.

The graph shows the response of HEK293T - hTRPM8 cells to Fluoxetine or Menthol. When fluoxetine (100 μM, blue bar) was applied first, there was no excitatory effect (point 1). Subsequently, when Menthol (500 μM, red bar) was applied, it showed an excitatory effect (point 2). The right panel corresponds to the left and shows the current-voltage curve of the voltage gradient from -100 mV to + 100 mV.

Figure S3

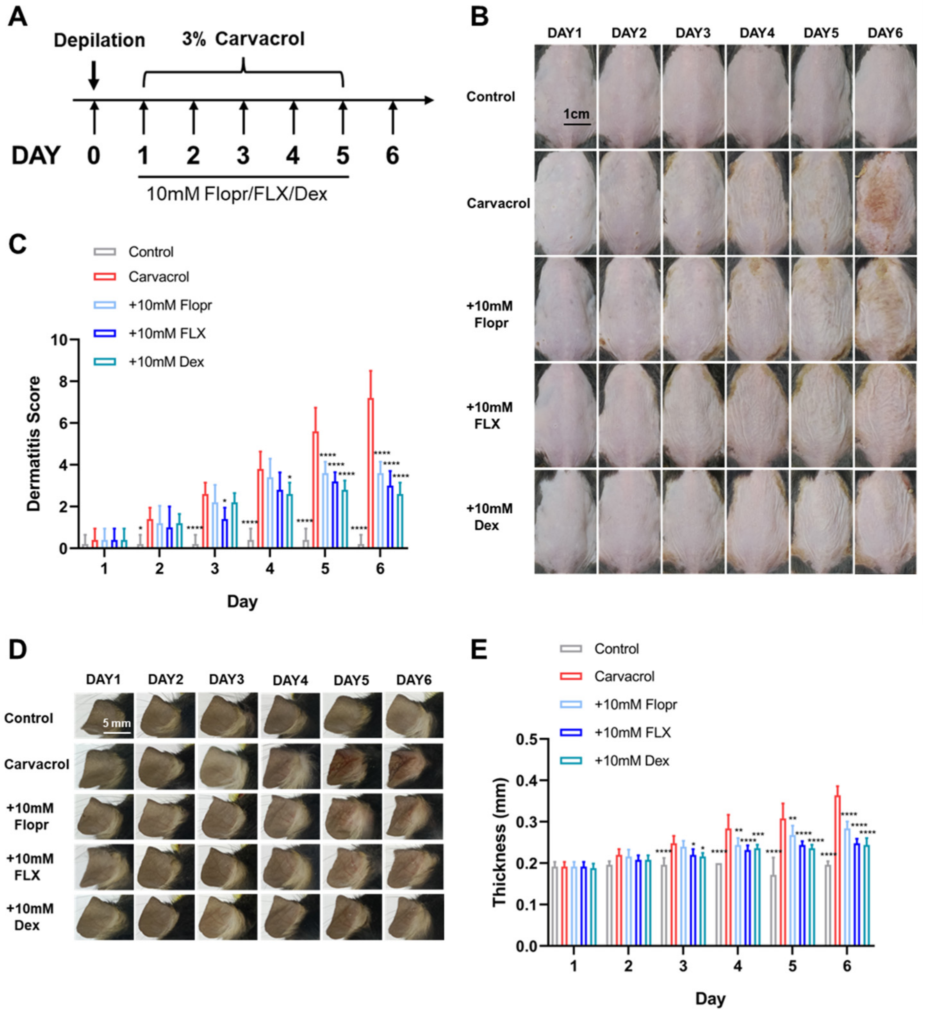

Figure S3. Drugs alleviate carvacrol-induced dermatitis and ear swelling. A, a flowchart depicting the establishment of carvacrol-induced mouse dermatitis and ear swelling and the administration of

fluoxetine (FLX), flopropione (Flopr) and dexamethasone (Dex). B, phenotypic features of mouse skin before and after 3% carvacrol in the presence of different drugs for six consecutive days. Scale bar = 1 cm. C, dermatitis scores from panel B (n = 5, *p <0 .05, ****p < 0.0001, followed by Dunnett's multiple comparisons test). All data were presented as the mean ± SD. D, observation for 6 consecutive days representative ear images of mouse. Scale bar = 5 mm. E, summary of ear thickness for carvacrol-induced ear swelling in mice treated with different drugs (n = 5, *p <0 .05, **p < 0.01, ***p <0 .001, ****p < 0.0001, followed by Dunnett's multiple comparisons test). All data were presented as the mean ± SD.
